# Accelerated Fe^III^/Fe^II^ redox cycle of Fenton reaction system using Pd/NH_2_-MIL-101(Cr) and hydrogen

**DOI:** 10.3906/kim-2008-34

**Published:** 2021-04-28

**Authors:** Zhong-Xing LIU, Xin LIU, Yong LI, Shi-Qian GAO

**Affiliations:** 1 Institute of Environmental Protection Application Technology, School of Environmental Science and Engineering, Tianping College of Suzhou University of Science and Technology, Suzhou University of Science and Technology, Suzhou, Jiangsu Province China; 2 Suzhou Mengli Environmental Technology Co., Ltd., Changshu National New & Hi-tech Industrial Development Zone Suzhou, Jiangsu Province China

**Keywords:** Fenton reaction, accelerated reduction of Fe^III^, hydrogen, palladium, NH_2_-MIL-101(Cr)

## Abstract

In this paper, a novel improvement in the catalytic Fenton reaction system named MHACF-NH_2_-MIL-101(Cr) was constructed based on H_2_ and Pd/NH_2_-MIL-101(Cr). The improved system would result in an accelerated reduction in Fe^III^, and provide a continuous and fast degradation efficiency of the 10 mg L^-1^ 4-chlorophenol which was the model contaminant by using only trace level Fe^II^. The activity of Pd/NH_2_-MIL-101(Cr) decreased from 100% to about 35% gradually during the six consecutive reaction cycles of 18 h. That could be attributed to the irreversible structural damage of NH_2_-MIL-101(Cr).

## 1. Introduction

The classic Fenton reaction has been widely applied in industrial wastewater treatment for the past 20 years because the refractory organics, such as phenols, aldehydes and halogenated hydrocarbons, could be efficiently degraded into low molecular weight organic acid, CO_2_ and H_2_O through this process under room temperature and normal pressure [1–5]. 

When the Fenton reaction is completed, the pH of effluent should be adjusted to 6~9 to meet the discharge quality of natural water. However, large amounts of iron sludge will be generated. That should be attributed to the ferrous salt that was continuously used to maintain the decomposition of hydrogen peroxide in order to produce hydroxyl radical (·OH).

Based on Equation (1) to (6) [6], more Fe^III^ will be produced in Fenton reaction. However, the regeneration of ferrous ions cannot be observed macroscopically. The iron sludge could not be reduced by incineration because it consisted of iron oxyhydroxides [7]. And it will take up lots of land if it is landfilled. Its proper disposal is closely related to the application and promotion of Fenton reaction in future. Thus, accelerating the reduction of Fe^III^ may be a useful method.

Fe^II^ + H_2_O_2_ →·OH + Fe^III^+ OH^-^ (1) 

Fe^III^ + H_2_O_2_ → Fe^II^+ H++ HO_2_· (2)

Fe^II^ + ·OH → OH^-^+ Fe^III^ (3)

H_2_O_2_ + ·OH → HO_2_·+ H_2_O (4)

Fe^III^ + HO_2_- → H++ Fe^II^ + O_2_ (5)

Fe^II^ + HO_2_- + H+→ Fe^III^ + H_2_O_2_ (6)

While previous studies utilised organic reductants like vitamin C, thioglycolic acid and tea polyphenols to achieve the above purpose [8–14], failure to control their quantity of accurately would result in a new organic pollution in effluent.

Granted there are many reductants, nevertheless, the use of hydrogen appears to the best choice due to it being an efficient inorganic reductant, the Fe^III^ could quickly be reduced by it without the problem of secondary organic pollution since its byproduct is just H_2_O as shown in Equation (7) [15]. This led to the development of the accelerated catalytic Fenton (ACF) system through the introduction of hydrogen and Pd^0^/Al_2_O_3_ into the classic Fenton system. The methyl tert-butyl ether could be continuously degraded using only1 mg L−1 Fe^II^ with the elimination of iron sludge [15,16].

[H] + [Fe(H_2_O)_5_ (OH)]^2+^→ [Fe (H_2_O)_6_]^2+^ (7)

Most of them will return back to the atmosphere immediately after it is supplied into the water. Therefore, it should be maintained at 0.1 MPa hydrogen partial pressure during the whole process which will cause huge waste [15,16].

With huge open metal sites and specific surface area, the metal-organic frameworks (MOFs) materials have gained more and more attention in the field of hydrogen storage [17–29], adsorption of pollutants [30–32], separation of gas component [33,34], monitoring pollutants [35,36] and hydrogen production [37,38]. And it could also be used as a carrier for noble metal catalysts, especially for Pd^0^ [39–43]. 

In our previous study, the hydrogen combination with MOFs material was first proposed to accelerate the reduction of Fe^III^ in Fenton reaction. A novel MOFs-hydrogen-accelerated catalytic fenton (MHACF) system was constructed using MIL-101(Cr) as the carrier of Pd^0^, leading to an extension in the retention time of H_2_ in solution. ·OH could be continuously generated by using only trace amounts of ferrous ion in that system. Although the MIL-101(Cr) will be gradually damaged during the reaction process [44,45]. These two papers would serve as a basis for further studies on the subject matter, with particular concentration on the reduction of iron sludge in Fenton system, and how improvements can be on suitable catalytic materials.

In this paper, H_2_ was still used to accelerate the Fe^III^/Fe^II^ cycling in Fenton reaction. Pd^0^, which is a common industrial catalyst and adsorbent and activator for hydrogen, (Equation (8)) [15,46] was chosen to enhance the retention of H_2_ in aqueous solution. The NH_2_-MIL-101(Cr), which could be stable in water and has acted as a novel material for hydrogen storage, applications in catalysis and the carrier for Pd^0^ [47–56], was chosen to be used in this paper. The reaction mechanism of this novel MHACF-NH_2_-MIL-101(Cr) system is depicted in Scheme 1. 

2Pd + H_2_ → 2Pd-[H] (8)

**Scheme:**
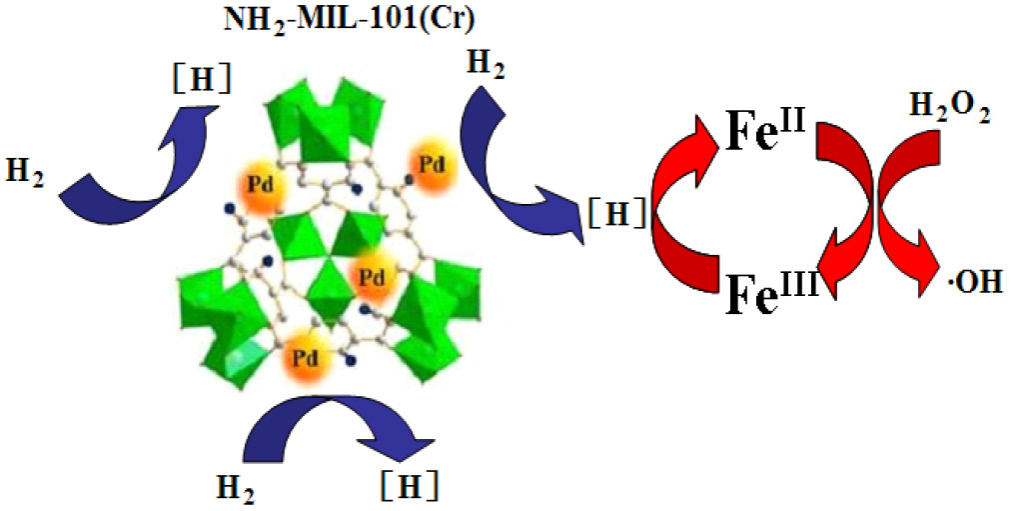


This work was to explore the cycling of Fe^III^/Fe^II^, and to investigate its degradation capacity and durability in this MHACF-NH_2_-MIL-101(Cr) system.

## 2. Materials and methods

### 2.1. Materials

All the chemical reagents were purchased from China National Pharmaceutical Group Corporation. They were of analytical grade (purity ≥ 99.0%) and used without further purification. All the aqueous solutions were prepared by ultra-pure water (Millipore Milli-Q system, resistivity > 18.2 MΩ). Ferrous ion, ferric ion and 4-chlorophenol solution were prepared by dissolving iron(II) sulfate heptahydrate, iron(III) chloride (anhydrous) and 4-chlorophenol in ultra-pure water. The initial pH was adjusted by sulfuric acid and sodium hydroxide. 

### 2.2. Synthetic procedures of the solid catalyst

The NH_2_-MIL-101(Cr) was produced by this procedure [57]. 0.72 g 2-aminoterephthalic acid, 1.6 g chromic nitrate nonahydrate, and 0.4 g NaOH were dispersed in 30 mL ultra-pure water and stirred for at least 10 min. This solution was added into a Teflon lined stainless steel autoclave. After the operation mentioned above, it was placed in a preheated oven in the condition of 150 °C. After 12 h reaction process, it was naturally cooled to 20 °C room temperature. Then, this precipitate was recovered by centrifugation. It was alternately washed by using N,N dimethylformamide, hot absolute alcohol, and ultra-pure water three times in room temperature. After the operation mentioned above, the solid product was dried in the condition of 80 °C in DZF-6930 vacuum drying oven (Shanghai Bluepard Instruments Co., Ltd, China). And it was also activated 150 min in the condition of 150 °C before it was utilised in it. 

The Pd/NH_2_-MIL-101(Cr) (Pd^0^ 0.5 wt% based on Agilent 720ES ICP-OES USA) was synthesised via a modification procedure, which was reported by Chen et al. [58]. 0.0188 g polyvinyl alcohol was dissolved in the condition of 95 °C. It was transferred into a three-necked flask, which contained 200 mg L^-1^ PdCl_2_ solution after it was cooled to 20 °C. After vigorous agitation for 1 h, the freshly prepared NaBH4 solution was drop wised into the flask. The Pd^0^ was generated by the reduction of PdCl_2_. The pH of aqueous solution was maintained at 7–8 by using HCl. Then 1 g activated MOFs material was added into the mixture immediately. It was agitated vigorously for 4 h. During the entire operation process, the whole flask was immersed into the ice-water mixture. Finally, the solid was collected and dried in the condition of 80 °C in a vacuum drying oven. It was also activated for 150 min in the same vacuum drying oven mentioned above in the condition of 150 °C before it was used in the reaction.

### 2.3. Characterisation of materials

All of the instruments used to characterise the materials including Fourier transform infrared spectroscopy (FT-IR), X-ray diffraction (XRD), Brunner-Emmet-Teller measurements (BET), X-ray photoelectron spectroscopy (XPS), scanning electron microscope (SEM) and transmission electron microscope (TEM) were same to those in our previous works [44,45].

### 2.4. The operation of the novel reaction system

The experiment was operated in a fume hood at normal temperature (20 ± 2 °C) and pressure. It was conducted in a 200 mL two-necked flat bottom flask equipped with magnetic stirrer. Before the experiment, the H_2_ produced by hydrogen generator (Beijing BCHP Analytical Technology Institute, China) was continuously supplied more than 5min to eliminate dissolved oxygen. Its purity was more than 99.999%. After the H_2_O_2_ was added into the flask, the experiment began. The escaping hydrogen was collected by the pipeline and sent to the roof by the fan. There was no fire source within 300 m around the exhaust vent. 

During the 6 consecutive reaction cycles, after the samples had been taken out at the end of each reaction cycle, H_2_O_2_ and 4-chlorophenol (4-CP) was immediately added into the system to maintain the initial concentration in the next reaction cycle at about 25 mM and 10 mg L^-1^, respectively.

Water samples were taken out by disposable syringe and filtered immediately by hydrophilic polyethersulfone filters which had a pore size of 0.45 μm. 

The filtrate was used for the concentration analysis of para-hydroxybenzoic acid (p-HBA), H_2_O_2_, 4-CP, ferrous ion, total Cr (Cr^3+^ and Cr^6+^, abbreviated as TCr) and total Pd (abbreviated as TPd).

### 2.5. Methods of sample analysis

The instrument and methods for quantitative analysis of pH, ferrous, low molecular weight organic acid, TCr, TPd, H_2_O_2_, p-HBA and 4-CP are the same as previous study [7,44,45,59,60]. Quantitative analysis of p-Hydroxybenzoic acid (p-HBA) and 4-CP was performed with LC-20AT high performance liquid chromatography (Shimadzu, Japan) equipped with a reversed phase Agilent ZORBAX Eclipse XDB-C18 column (Agilent, USA) (4.6 mm × 150 mm, 5 μm). A SPD-M20A detector was used for the analysis. The concentration of Cl- was measured by DIONEX ICS-900 (DIONEX, USA) coupled with a conductivity detector, IonPac AS23 Anion-Exchange Column and IonPac AG23 Guard Column (DIONEX, USA) at 24 ℃. The eluent, containing 4.5 mM Na_2_CO_3_ + 0.8 mM NaHCO3, was pumped at a flow rate of 1.0 mL min^-1^. The suppresion current and injection volume were set to 46 mA and 10 μL, respectively. The concentration of iminodiacetate, formate and acetate was measured by DIONEX ICS-1100 (DIONEX, USA) coupled with a conductivity detector, IonPac AS11-HC anion-exchange column and IonPac® AG11-HC guard column at 24 ℃. The eluent of KOH was pumped at a flow rate of 1.0 mL min^-1^. The elution program was as follows: 0～17 min, 0.8 mM KOH; 17～52 min, 30 mM KOH; 52～55min, 0.8 mM KOH. The suppresion current and injection volume were set to 120 mA and 150 μL respectively. pH value was determined by pHS-3C pH meter (Shanghai Precision & Scientific Instrument Co. Ltd, Shangai, China). The error bars in all of the figures represented the standard deviation after three measurements

## 3. Results and discussion

### 3.1. Characterisation of the materials

The characterisation of the MOFs materials can be seen in the supplementary materal. 

### 3.2. Transformation of iron valence in the reaction system

**Figure 1:**
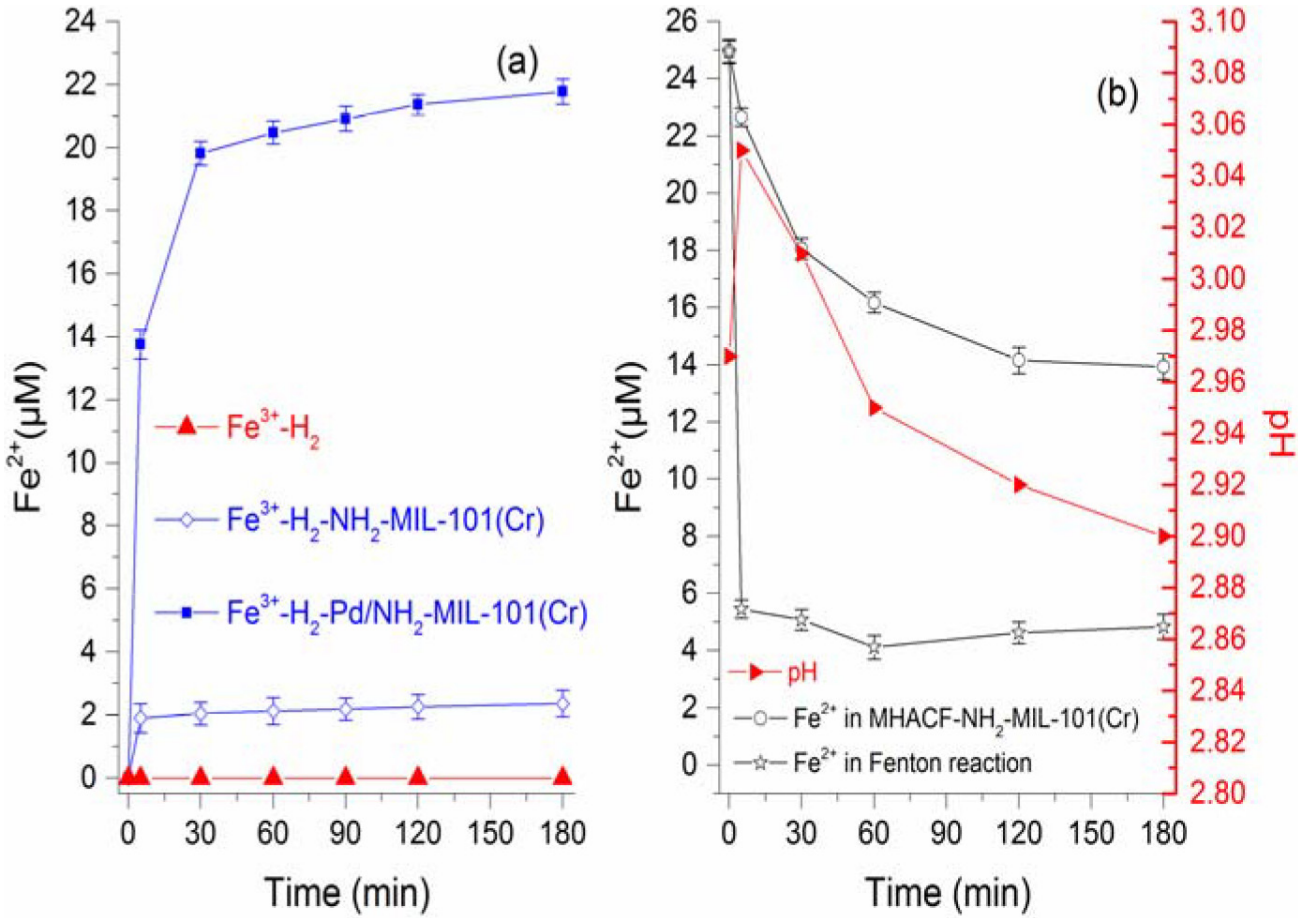
(a) Variation of ferrous ion in Fe3+-H2-Pd/NH2-MIL-101(Cr), Fe3+-H2-MIL-101(Cr), and Fe3+-H2 system, respectively (the initial aqueous solution pH was about 2.5); (b) variation of pH and ferrous ion in MHACF-NH2-MIL-101(Cr) system, and variation of ferrous ion in Fenton system. Except for the parameters investigated, other initial parameters were NH2-MIL-101(Cr) 2 g L-1, Fe3+ 25 μM, H2 85 mL min-1, H2O2 25 mM, Pd/NH2-MIL-101(Cr) 2 g L-1, Fe2+ 25 μM and initial pH 3.

The transformation of iron valence in H_2_ without using MOFs system, H_2_-MOFs system, and H_2_-Pd/MOFs system was shown in Figure 1(a). Ferrous ions could not be detected in the H_2_ without using MOFs system. That means the reduction of Fe^III^ could be negligible because H_2_ is insoluble in water. Ferrous ions increased from 0 to 21.78 μM, and to 2.26 μM in H_2_-Pd/MOFs and H_2_-MOFs systems respectively. This means Fe^III^ could be reduced by hydrogen. And the reduction of Fe^III^ was enhanced significantly by using NH_2_-MIL-101(Cr) and Pd^0^ compared with that of the introduction of only NH_2_-MIL-101(Cr). That means the retention time of H_2_/[H] in aqueous solution was extended (Equation (8) and Equation (9)) [15].

Pd-[H] + Fe^III^ → Pd + Fe^II^ + H^+^ (9)

As seen in Figure 1(b), the solution pH ranged from 2.9 to 3.05, showing that Equation (8) and Equation (9) existed in this system. Fe2+ decreased gradually from 24.96 μM to 13.94 μM. It decreased from 24.94 μM to 5.46 μM within five minutes after the start of the reaction in the classic Fenton system. Then it decreased gradually to 4.84 μM in the remaining 175 min. This means the reduction of Fe^III^ was enhanced by hydrogen in this novel system (Equation (1), Equation (3) and Equation (6)). Therefore, the degradation ability of MHACF-NH_2_-MIL-101(Cr) could be improved using only trace amounts of Fe due to the rapid cycling of Fe^III^/Fe^II^.

### 3.3. Hydroxyl radicals in the system

**Figure 2:**
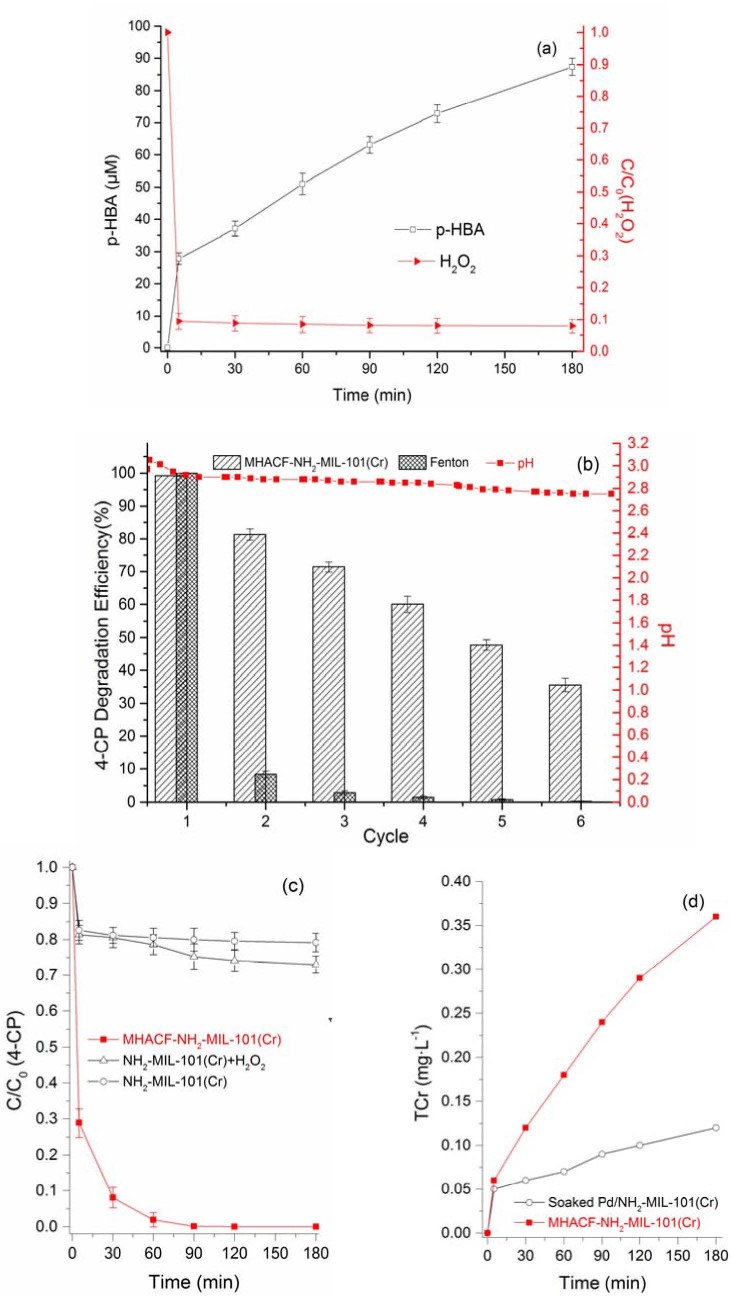
(a) Variation of p-HBA and H2O2 in MHACF-NH2-MIL-101(Cr) system (BA was used as ·OH scavenger); (b) 4-CP degradation efficiency of 6 consecutive reaction cycles in Fenton and MHACF-NH2-MIL-101(Cr) system, respectively. Variation of pH in MHACFNH 2-MIL-101(Cr) during 6 consecutive reaction cycles (each reaction cycle was 180 min); (c) the control test of 4-CP; (d) variation of TCr in H2SO4 solution (without H2, 4-CP, Fe2+ and H2O2) and MHACF-NH2-MIL-101(Cr) system (without 4-CP), respectively. Except for the parameters investigated, other initial parameters were BA 1.8 g·L-1, Fe2+ 25 μM, H2O2 25 mM, Pd/NH2-MIL-101(Cr) 2 g L-1, H2 85 mL min-1, and pH 3.

The H_2_O_2_ in the novel system decreased from 25.06 mM to 2.35 mM in the first 5 min, as shown in Figure 2(a). Then, it decreased gradually to 1.98 μM in the remaining 175 min. Besides, the benzoic acid (BA) was used as the quencher of ·OH in this work. The quenched product was p-HBA [59]. The p-HBA increased rapidly from 0 to 87.39 μM after the whole reaction process. Therefore, it could be verified that the ·OH derived from the decomposition of H_2_O_2_ existed in this novel system.

### 3.4. The robustness of solid catalyst in the system

As a raw material for disinfectants, leather preservatives and insecticides, the 4-CP is one of representative refractory organic pollutant in both developing and developed countries but due to it being a toxic pollutant, it is banned in developed countries [61,62]. In this paper, it was chosen as the model contaminant. Previous studies showed that direct oxidation and stripping of O_2_, and the coagulation sedimentation of iron salt could be negligible in the removal of 4-CP, while it could be rapidly degraded in the Fenton system [7].

Results obtained in Figure 2(b) shows that in the 1^st^ reaction cycle of the classic Fenton reaction, 4-CP could be degraded thoroughly, though the degradation efficiency in the 2^nd^ 3h reaction cycle rapidly decreases to 8.5%. The degradation efficiency maintained this downward trend in the rest 4 cycles. The degradation efficiency was less than 1% after completing the whole 6 reaction cycles. From Equation(1) to Equation(6), the ·OH was insufficient in the 2^nd^, 3^rd^, 4^th^, 5^th^ and 6^th^ cycle. This may be attributed to the faster consumption and slower regeneration of Fe^II^.

In contrast, 4-CP could be eliminated thoroughly in 120 min in the 1^st^ reaction cycle of MHACF-NH_2_-MIL-101(Cr) system and it could maintain more than 35% degradation efficiency after 6 consecutive reaction cycles by using only trace amount of Fe^II^/Fe^III^. It means ·OH was comparatively sufficient during the whole 6 reaction cycles and this could be attributed to the faster regeneration of Fe^II^ accelerated by using hydrogen and Pd/NH_2_MIL-101(Cr) (Equation(8), Equation(9)) [15].

The pH gradually decreases from 2.97 at the beginning of the reaction to 2.75 after 6 consecutive reaction cycles. This phenomenon reflects the fact that the Fe^II^ regenerated rapidly by the reduction of Fe^III^ (Equation (9)).

In the 1^st^ cycle of this novel system, 0.56 mg L^-1^ 4-chlorobenzene-1,2-diol and 0.47 mg L^-1^ cis-2-butenedioic acid was detected at 110 min., 0.42 mg L^-1^ cis-2-butenedioic acid was detected at 160 min. The p-benzoquinone and hydroquinone could not be detected. This result was similar to findings from other studies [7,44,45,63].

Phenols could be removed by the adsorption process of MOFs [64]. According to the BET test, the specific surface area of NH_2_-MIL-101(Cr), before it was utilised in the reaction, was 1698.99 m2 g-1. Less than 20% 4-CP could be removed by the adsorption of MOFs, as shown in Figure 2(c). The difference in removal efficiency between NH_2_-MIL-101(Cr) system and NH_2_-MIL-101(Cr) + H_2_O_2_ system was not significant. The generation of ·OH in NH_2_-MIL-101(Cr) + H_2_O_2_ system could be negligible. Therefore, the role of this MOFs material in the MHACF-NH_2_-MIL-101(Cr) system was hydrogen storage material and carrier of Pd^0^.

As shown in Figure 2(b), the degradation efficiency in MHACF-NH_2_-MIL-101(Cr) system was 100% (1^st^ cycle), 81.32% (2^nd^ cycle), 71.42% (3^rd^ cycle), 60.08% (4^th^ cycle), 47.48% (5^th^ cycle) and 35.62% (final cycle), respectively. Combined with the results depicted in Figure 2(d), the TCr increased gradually from 0 to 0.36 mg·L^-1^ during the 180 min reaction. The variation of TCr in H_2_SO4 aqueous solution was similar with our previous work [44,45]. The Pd was not detected in both solutions. Based on these results, it could be speculated that NH_2_-MIL-101(Cr) was damaged gradually after the long-term operation of this novel system.

**Figure 3:**
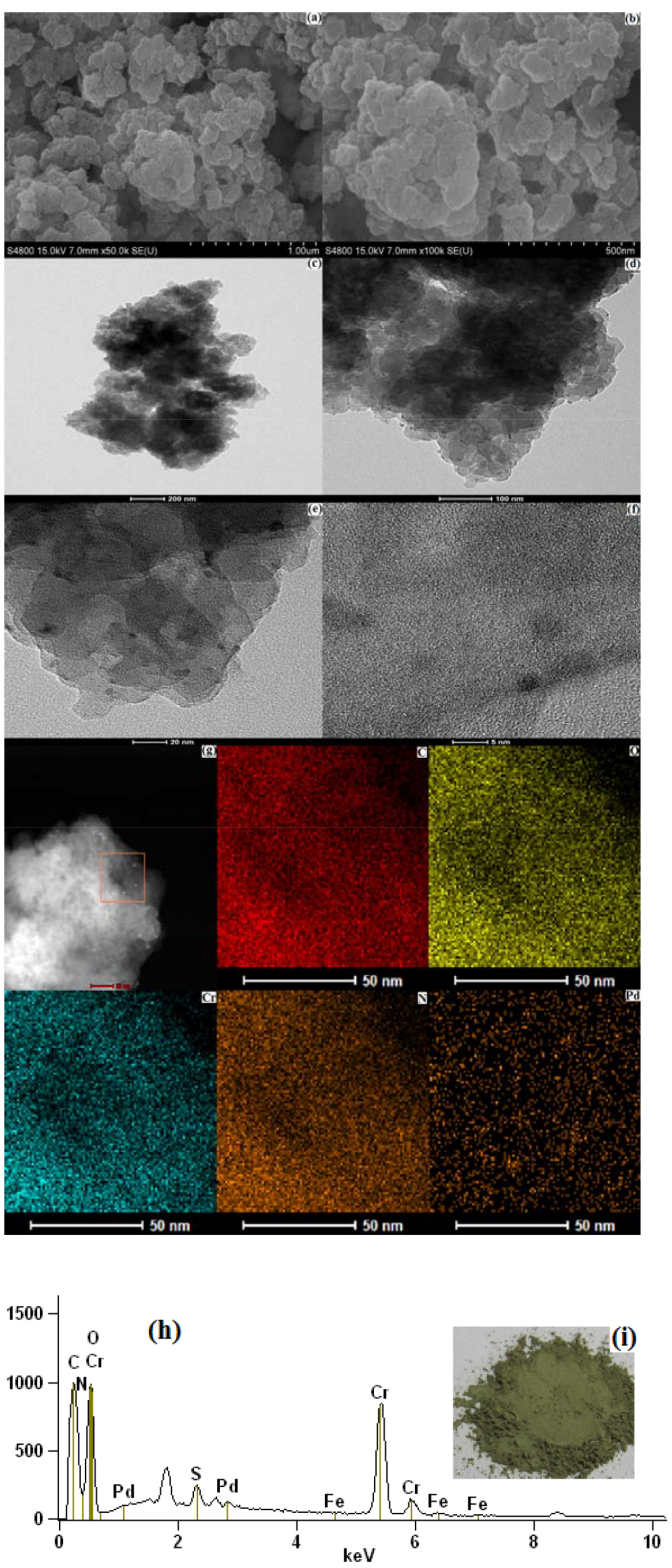
Characterisation of the residue in MHACF-NH2-MIL-101(Cr) system after 6 consecutive reaction cycles (without 4-CP): (a) the SEM photograph (1 μm scale); (b) the SEM photograph (500 nm scale); (c) the TEM photograph (200 nm scale); (d) The TEM photograph (100 nm scale); (e) the TEM photograph (20 nm scale); (f) the TEM photograph (5 nm scale); (g) element mappings images; (h) the EDX photograph; (i) the appearance of residue. Each reaction cycle was 180 min.

As revealed in Figure 3, the morphological change in Pd/NH_2_-MIL-101(Cr) provided further direct evidence. As depicted in Figure 3(h), Figure 3(g) and Figure S2, the EDX spectrum, the element mapping images and the XPS signal intensiveness of the dark yellow green powder (shown in Figure 3(i)) which was the residue collected after the 6 reaction cycles, revealed that the C, N, Cr and Pd were still distributed in it, while its surface area was lower to only 841.71 m2 g-1. The results displayed in Figures 3(a) and 3(b) shows that the average diameter of single Pd/NH_2_-MIL-101(Cr) particle was less than 300 nm. The appearance of the particles was more irregular compared with that exhibited in Figure S6(a) and Figure S6(b). Figure 3(c) to Figure 3(f) shows an octahedral structure which is the representative octahedral structure of NH_2_-MIL-101(Cr) was not present in the residue when compared to what is exhibited in Figure S6(c) to Figure S6(f). Also, according to the XRD pattern of the residue shown in Figure S4(d), the intensiveness of the detection signal was significantly weaker after the 6 cycle reactions. Furthermore, according to XPS spectrum of Pd element depicted in Figure S3(d), the intensiveness of the residue increased significantly. This may be attributed to the Pd^0^ loaded on the surface of this MOFs material being shed off during the reaction with the structural damage of NH_2_-MIL-101(Cr) and the reduction of surface area, and then was gathered in aqueous solution. It was similar to our previous research results from MHACF-MIL-101(Cr) [44,45]. As established by the hard/soft acid/base principle, a more stable framework could be formed by high-valent metal cations by stronger coordination bonds. MOFs containing a higher oxidation state metal or a metal clusters tend to be more stable in acids due to the stronger coordination bonds [65,66]. Therefore, the MOFs consisting of the group 4 metal cations of the periodic table may be a new research direction.

## 4. Conclusion

With using trace amount of ferrous salt, the oxidative degradation capacity of novel MHACF-NH_2_-MIL-101(Cr) system was much higher than that of Fenton system under normal pressure and temperature. The regeneration of Fe^II^ could be significantly accelerated by using hydrogen gas and solid catalyst Pd/NH_2_-MIL-101(Cr). Under the premise that the hydroxyl radical could be continuously produced in this novel system, the yield of iron sludge could be reduced. However, the structure of NH_2_-MIL-101(Cr) would be damaged in acidic condition which led to the leaching of chromium ions. In the future, more stable MOFs materials for the storage and activation of hydrogen are recommended to solve the above questions thoroughly. The idea behind this novel system offers new way for the research and development of significantly improved the application of Fenton reaction. This work is the beginning of a series of research work in future. We hope that scholars and engineers could focus on the improvement of Fenton reaction and explore and improve the suitable catalytic materials.
